# The Microbiota–Metabolism Axis: Mechanistic Insights and Translational Targets Across Diabetes and Gastrointestinal Disorders

**DOI:** 10.1155/ije/7761643

**Published:** 2026-11-09

**Authors:** Adnan Akif, Nigar Sultana, Ishtyaque Mikrani

**Affiliations:** ^1^ Department of Physiology & Cell Biology School of Medicine University of Nevada Reno Nevada USA unlv.edu; ^2^ Sir Salimullah Medical College Dhaka Bangladesh; ^3^ Department of Pharmacy National Model College for Advance Learning Tribhuvan University Kathmandu Nepal tribhuvan-university.edu.np

**Keywords:** diabetes, fecal microbiota, gut microbiota, inflammatory bowel disease, metabolite

## Abstract

The gut microbiota (GM) modulates host energy balance, glucose–lipid metabolism, and enteroendocrine signaling through metabolite–receptor axes that include short‐chain fatty acids (SCFAs) acting on FFAR2/FFAR3 (GPR43/GPR41), microbially transformed bile acids signaling via FXR/TGR5, and amino‐acid/choline‐derived compounds such as imidazole propionate (ImP) and trimethylamine N‐oxide (TMAO). These pathways regulate incretin biology (GLP‐1/PYY), insulin sensitivity, hepatic glucose output, adipose lipolysis, and appetite, thereby shaping risk and progression of Type 2 diabetes mellitus (T2DM), obesity, inflammatory bowel disease (IBD), irritable bowel syndrome (IBS), and related conditions. Here, we synthesize mechanistic and translational evidence, highlight bidirectional drug–microbiome interactions (GLP‐1 receptor agonists), and review interventions spanning diet, pre/probiotics, fecal microbiota transplantation (FMT), and pharmacologic targeting of bile‐acid and SCFA pathways. Together, these findings support precision strategies that profile GM–metabolite signatures (e.g., SCFAs, secondary bile acids, and ImP TMAO) to guide prevention and therapy in metabolic and gastrointestinal disease.

## 1. Introduction

The human gastrointestinal tract is home to a dense and dynamic community of microorganisms, collectively known as the gut microbiota (GM), which coevolved with the host in a symbiotic relationship [1, 2]. For decades, this microbial community was viewed primarily through the lens of digestion and pathogen exclusion [3, 4]. However, a pattern has emerged, recasting the GM as a vital, metabolically active endocrine organ integral to host physiology. The collective microbial genome, or microbiome, contains a set of genes that is orders of magnitude larger than the human genome, endowing it with a vast enzymatic capacity to metabolize dietary components, such as complex plant fibers, that are otherwise indigestible by the host [5, 6]. This metabolic activity generates a rich milieu of bioactive small molecules that are absorbed into systemic circulation, where they function as signaling molecules that influence the function of distal tissues and organs [7, 8].

The molecular interactions of this host–microbiome crosstalk are mediated by specific classes of microbial metabolites. Among these, short‐chain fatty acids (SCFAs), secondary bile acids (BAs), and derivatives of amino acid and lipid metabolism have emerged as critical regulators of host energy homeostasis [9, 10]. Dysregulation in the production and signaling of these metabolites is now mechanistically linked to the pathophysiology of a wide spectrum of noncommunicable diseases, most notably Type 2 diabetes mellitus (T2DM), obesity, inflammatory bowel disease (IBD), and irritable bowel syndrome (IBS) [8, 11–13]. This evolution in understanding suggests that a focus on “functional dysbiosis”—the alteration of key metabolic outputs of the microbiome—may be more clinically relevant than merely cataloging changes in microbial composition [14, 15]. The metabolic signature of a patient’s microbiome, such as elevated levels of detrimental metabolites or a deficit in beneficial ones, may better predict disease phenotype and progression than the presence or absence of a particular bacterial species.

This review aims to synthesize the current mechanistic understanding of how key microbial metabolites modulate host metabolic signaling pathways. It will examine the clinical evidence linking these pathways to T2DM and major GI disorders and critically evaluate emerging therapeutic strategies designed to target the microbiota–metabolism axis, including diet, pharmacotherapies, and fecal microbiota transplantation (FMT). Finally, this review will provide a forward‐looking perspective on the translation potential of these findings for developing precision medicine approaches to prevent and manage complex metabolic and gastrointestinal diseases.

## 2. Gut Microbial Metabolites and Host Metabolic Signaling Pathways

The GM communicates with the host through a diverse array of metabolites that activate specific host receptors and signaling cascades. Three axes have been identified as particularly crucial in the regulation of glucose homeostasis, insulin sensitivity, and inflammation: the SCFA–Free Fatty Acid Receptor 2/3 (FFAR2/FFAR3) axis, the BA–FXR/TGR5 axis, and pathways involving pathogenic metabolites derived from amino acid and lipid precursors.

### 2.1. The SCFA–FFAR2/FFAR3 Axis

Indigestible dietary fibers, such as cellulose, resistant starch, and polysaccharides found in legumes, serve as the primary substrates for microbial fermentation in the colon [16]. Specialized anaerobic bacteria, including members of the genera *Faecalibacterium*, *Roseburia*, and *Bifidobacterium*, metabolize these complex carbohydrates to produce SCFAs, principally acetate, propionate, and butyrate, as shown in Figure 1 [17]. These SCFAs are not merely metabolic byproducts; they are potent signaling molecules that activate a class of G‐protein‐coupled receptors, most notably FFAR2 (also known as GPR43) and FFAR3 (GPR41). These receptors are highly expressed on the surface of enteroendocrine L‐cells (Figure 1), which are interspersed throughout the intestinal epithelium and are responsible for producing key metabolic hormones [18].

**FIGURE 1:**
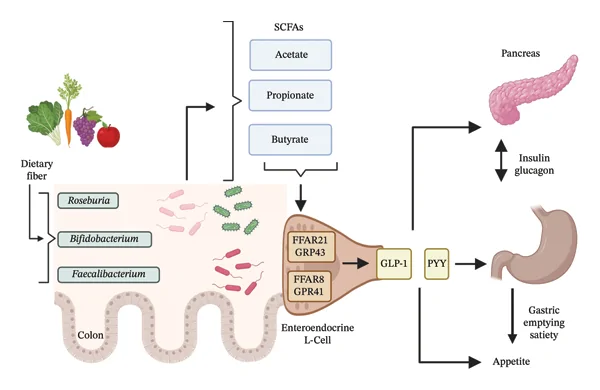
SCFA–GPR41/43–incretin pathway. Indigestible dietary fiber reaches the colon, where commensal genera (*Roseburia*, *Bifidobacterium*, *Faecalibacterium*) ferment it into short‐chain fatty acids (SCFAs)—acetate, propionate, and butyrate. SCFAs stimulate enteroendocrine L‐cells by activating free‐fatty‐acid receptors FFAR2 (GPR43) and FFAR3 (GPR41), leading to secretion of the incretins GLP‐1 and PYY. GLP‐1 acts on the pancreas to enhance insulin and suppress glucagon, while GLP‐1 and PYY together slow gastric emptying, increase satiety, and reduce appetite. Arrows indicate the direction of signaling from diet ⟶ microbiota ⟶ SCFAs ⟶ L‐cell receptors ⟶ hormonal outputs and metabolic effects.

Activation of FFAR2 and FFAR3 on L‐cells by SCFAs triggers the secretion of glucagon‐like peptide‐1 (GLP‐1) and peptide YY (PYY), two crucial incretin hormones that regulate glucose homeostasis and appetite [19]. GLP‐1 enhances glucose‐stimulated insulin secretion (GSIS) from pancreatic *β*‐cells, suppresses glucagon release, slows gastric emptying, and promotes satiety [20]. PYY primarily acts to reduce appetite and food intake [21]. Direct causal evidence for this SCFA‐incretin link comes from preclinical studies. For instance, scientists have demonstrated in a diabetic mouse model that targeted colonic delivery of acetate, via a specially designed water‐soluble cellulose acetate (WSCA), significantly improved glucose tolerance [22]. This metabolic benefit was directly associated with elevated circulating levels of both GLP‐1 and PYY, confirming that colonic SCFAs can systemically improve glycemic control by augmenting incretin hormone secretion. In a mouse model of glucocorticoid‐induced metabolic syndrome, Zhang et al. (2024) observed that the development of hyperglycemia and insulin resistance was preceded by profound gut dysbiosis, characterized by a loss of SCFA‐producing bacteria such as *Lachnospiraceae* [23]. This compositional shift resulted in markedly reduced levels of acetate and propionate in the gut, which in turn led to lower circulating GLP‐1. Mechanistically, this hormonal deficit was linked to decreased colonic expression of the FFAR3 (GPR41) receptor, solidifying the critical role of the entire GM–SCFA–FFAR3–GLP‐1 pathway in maintaining metabolic health.

### 2.2. The BA–FXR/TGR5 Axis

BAs are another class of molecules whose biological activity is profoundly shaped by the GM. The liver synthesizes primary BAs, primarily cholic acid and chenodeoxycholic acid, which are conjugated to glycine or taurine and secreted into the duodenum to aid in lipid digestion. While a majority of these are reabsorbed in the ileum, a significant portion reaches the colon, where they are subject to extensive biotransformation by microbial enzymes [24]. As illustrated in Figure 2, bacteria deconjugate, dehydroxylate, and epimerize primary BAs, generating a complex pool of over 50 different secondary BAs, including deoxycholic acid, lithocholic acid, and ursodeoxycholic acid [25]. This microbial processing is critical, as primary and secondary BAs have distinct affinities for host receptors and thus exert different physiological effects.

**FIGURE 2:**
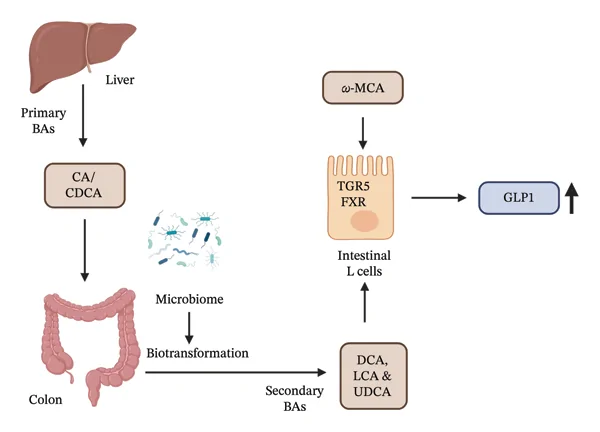
Bile‐acid signaling through FXR/TGR5 drives GLP‐1 release. The liver synthesizes primary bile acids (BAs)—cholic acid (CA) and chenodeoxycholic acid (CDCA)—which enter the intestine and reach the colon, where the microbiome biotransforms them into secondary BAs, including deoxycholic acid (DCA), lithocholic acid (LCA), and ursodeoxycholic acid (UDCA). Microbiota‐dependent secondary BAs (e.g., *ω*‐muricholic acid and *ω*‐MCA) activate Takeda G‐protein‐coupled receptor 5 (TGR5) and the nuclear receptor farnesoid X receptor (FXR) on intestinal L‐cells, stimulating glucagon‐like peptide‐1 (GLP‐1) secretion. Arrows indicate the sequential flow from hepatic bile acid (BA) synthesis to microbial conversion of BAs, followed by BA‐mediated receptor activation on L‐cells, which ultimately increases circulating GLP‐1.

Secondary BAs function as signaling hormones that regulate glucose and energy metabolism by activating two key receptors: the nuclear receptor FXR and the membrane‐bound Takeda G‐protein‐coupled receptor 5 (TGR5) [26]. Like the SCFA receptors, TGR5 is expressed on intestinal L‐cells, and its activation is a potent stimulus for GLP‐1 secretion (Figure 2). The indispensable role of the microbiota in this process was elegantly demonstrated by Wang et al. [27]. Using antibiotic‐treated and germ‐free mice, they showed that the normal postprandial surge in GLP‐1 following a lipid meal was completely abolished in the absence of a GM. This incretin response could be restored by FMT. Further mechanistic work identified specific microbiota‐dependent secondary BAs, such as *ω*‐muricholic acid, as the key signaling molecules that activate TGR5 on L‐cells to drive GLP‐1 release [28]. This research pinpoints the microbiota–BA–TGR5 axis as an essential component of postprandial glucose regulation, acting in concert with the SCFA pathway to fine‐tune incretin hormone dynamics.

### 2.3. Pathogenic Metabolites: Imidazole Propionate (ImP) and Trimethylamine N‐Oxide (TMAO)

While the fermentation of fiber and metabolism of BAs generally produce beneficial signals, an altered microbial community can also generate metabolites that are detrimental to host metabolic health. This represents a “dark side” of microbial metabolism, where the metabolic output of a dysbiotic gut actively drives disease pathology. The metabolic landscape of T2DM can thus be viewed as a net balance, or imbalance, between these protective and pathogenic signals. The pathophysiology of metabolic disease is not merely the absence of beneficial metabolites like SCFAs but the simultaneous overproduction of harmful ones, creating a multi‐pronged assault on host metabolic control systems.

#### 2.3.1. ImP and Insulin Resistance

ImP is a microbial metabolite produced from the fermentation of the dietary amino acid histidine. Mounting evidence has implicated ImP as a direct driver of insulin resistance. In a large, multi‐center European study, Molinaro et al. (2020) found that serum levels of ImP were significantly elevated in individuals with prediabetes and T2DM compared to normoglycemic controls [29]. Critically, this association was not due to differences in dietary histidine intake but was instead linked to an altered microbial ecology. Individuals with high ImP levels were more likely to harbor a low‐diversity “Bacteroides 2” enriched in the microbial genes required for ImP biosynthesis. The molecular mechanism by which ImP impairs insulin action was elucidated by Koh et al. [30]. Their work demonstrated that ImP directly inhibits the insulin signaling cascade in host cells. ImP activates the kinase p38*γ*, which in turn leads to the hyperactivation of the mechanistic target of rapamycin complex 1 (mTORC1)/S6K1 pathway. Activated S6K1 then phosphorylates and inhibits insulin receptor substrate 1, a critical upstream node in the insulin signaling pathway, thereby blunting the cell’s response to insulin. This mechanism has direct clinical implications; the same study showed that administration of ImP to mice blunted the glucose‐lowering effect of metformin, the first‐line therapy for T2DM. In humans with T2DM, higher circulating ImP levels correlated with a poorer glycemic response to metformin treatment [30]. This positions ImP as a microbially produced uremic toxin that not only contributes to the pathogenesis of T2DM but also interferes with its treatment.

#### 2.3.2. TMAO and *β*‐Cell Dysfunction

TMAO is another well‐studied microbial metabolite linked to adverse health outcomes, particularly cardiovascular disease [31]. It is generated in a two‐step process: gut microbes metabolize dietary precursors rich in choline and L‐carnitine (e.g., red meat, eggs) into trimethylamine (TMA), which is then absorbed and oxidized to TMAO by flavin‐containing monooxygenase enzymes in the liver. Recent research has now uncovered a direct role for TMAO in the pathogenesis of T2DM by targeting pancreatic *β*‐cells. A study by Kong et al. revealed that TMAO, at concentrations equivalent to those found in individuals with diabetes, directly impairs GSIS from both mouse and human pancreatic islets [32]. Chronic elevation of TMAO in mice led to impaired glucose tolerance, reduced *β*‐cell mass, and worsened glycemic control. The underlying mechanism involves the induction of endoplasmic reticulum (ER) stress and activation of the NOD‐, LRR‐ and pyrin domain‐containing protein 3 (NLRP3) inflammasome within the *β*‐cell [31]. This inflammatory cascade disrupts intracellular calcium signaling, a process essential for insulin granule exocytosis, and over time promotes *β*‐cell dedifferentiation and apoptosis. These findings identify TMAO as a gut‐derived *β*‐cell toxin that contributes to the progressive decline in insulin secretory capacity that is a hallmark of T2DM.

## 3. Microbiota–Endocrine Crosstalk: A Hormonal Symphony

The evidence synthesized above firmly establishes the GM as an upstream regulator of the host’s endocrine system. By producing SCFAs and secondary BAs, the microbial community directly modulates the secretory function of intestinal L‐cells, thereby controlling the release of GLP‐1 and PYY [33]. This positions the microbiome as a pivotal determinant of the “incretin effect”—the physiological phenomenon whereby oral glucose administration elicits a significantly greater insulin response than an equivalent intravenous glucose load. A healthy, fiber‐fermenting microbiota is therefore essential for a robust incretin response and efficient postprandial glucose disposal.

This intricate relationship also creates the potential for bidirectional interactions with modern diabetes therapies, particularly incretin‐based drugs like GLP‐1 receptor agonists. While these drugs are highly effective, they also exert significant effects on the gut environment by slowing gastric emptying and intestinal motility, which can in turn alter the composition and function of the resident microbiota. Consistent with this concept, a recent systematic review of 38 preclinical and human studies found that GLP‐1 analogs and agonists can alter gut microbial composition, richness, and diversity, although the clinical significance and causal contribution of these changes remain to be established [34]. This raises the possibility of a feedback loop where the drug modifies the microbiome, and the altered microbiome then influences the host’s long‐term response to the therapy. This interplay highlights an emerging field of “pharmacomicrobiomics,” where an individual’s gut microbiome is recognized as a key factor influencing drug efficacy, metabolism, and side‐effect profile. The finding by Koh et al. that the microbial metabolite ImP can blunt the action of metformin is a prime example of this phenomenon [30]. This suggests that profiling a patient’s microbial or metabolic signature could one day be used to predict their response to a given therapy, paving the way for a more personalized approach to diabetes management. For instance, a patient with very high baseline ImP levels might be a poor candidate for metformin as a first‐line agent, potentially guiding clinicians toward an alternative therapy.

## 4. Clinical Manifestations of a Dysbiotic Metabolism

The dysregulation of these microbiota–metabolite axes manifests clinically in a range of metabolic and gastrointestinal disorders. The underlying mechanisms are often shared, creating a web of interconnected pathophysiology that links the gut to systemic health.

### 4.1. T2DM and Obesity

T2DM and obesity are best understood as disorders of altered microbial function rather than isolated taxonomic change. Recent biomarker‐oriented reviews show that T2DM shares with other chronic inflammatory disorders a disruption in the host handling of bacteria‐derived metabolites, while human studies in prediabetes and T2DM consistently identify low microbial gene richness, depletion of beneficial fermentative functions, and enrichment of metabolite‐producing microbial configurations linked to insulin resistance and metabolic inflammation [35]. This function‐first model is important because the diabetic microbiome is not defined only by which organisms are present but also by which metabolites they produce, including SCFAs, BAs, ImP, and TMAO [31, 33]. This function‐centered interpretation is also reflected in recent expert translational assessments of the diabetes microbiome field, which emphasize the need to move beyond taxonomic associations toward microbial functions, metabolites, causal mechanisms, and clinically actionable biomarkers [36].

A central mechanism involves loss of fiber fermentation and impaired SCFA signaling. Acetate, propionate, and butyrate regulate enteroendocrine L‐cell activity through FFAR2/FFAR3‐dependent signaling, augment GLP‐1 and PYY secretion, and thereby influence satiety, postprandial insulin release, and glucose homeostasis [33]. Butyrate is also a major oxidative fuel for colonocytes and strengthens epithelial integrity by facilitating tight‐junction assembly and by inhibiting histone deacetylases, linking microbial fermentation directly to barrier maintenance and inflammatory tone [37]. The translational relevance of this pathway is increasingly supported by intervention studies. In a randomized controlled trial (RCT) in prediabetes, a legume‐enriched calorie‐restricted diet produced greater reductions in HbA1c and low‐density lipoprotein (LDL) cholesterol than calorie restriction alone and was accompanied by increased fiber‐degrading species and altered bile‐acid and amino‐acid metabolites [38]. Similarly, targeted colonic acetate delivery through WSCA increased circulating GLP‐1 and PYY and improved glucose intolerance in db/db mice [22].

Bile‐acid remodeling represents a second major pathway linking microbiota to diabetic physiology. Microbial deconjugation and downstream transformation of primary BAs reshape the relative abundance of ligands for FXR and TGR5, thereby influencing enterohepatic feedback, GLP‐1 secretion, hepatic glucose production, and whole‐body energy homeostasis [27]. This is mechanistically important because BAs are not passive digestive surfactants; they are host–microbe signaling molecules that integrate nutrient sensing with metabolic regulation. Consequently, dysregulated bile‐acid pools should be examined as functional mediators and candidate biomarkers of metabolic disease rather than as secondary epiphenomena.

A further layer of pathogenic specificity is provided by harmful amino‐acid‐ and choline‐derived metabolites. In a large multicenter human cohort, circulating ImP was elevated in prediabetes and diabetes, associated with low bacterial gene richness and the Bacteroides 2 enterotype, and appeared to reflect altered microbial histidine metabolism rather than increased histidine intake alone [29]. More recent kinetic work has reinforced the importance—and complexity—of this pathway by showing that dietary histidine can be converted into ImP and that the GM plays a critical role in regulating this conversion in both mice and humans [39]. TMAO provides an additional example of a microbiota‐derived metabolite with direct pancreatic effects. It has now been shown to impair GSIS in mouse and human islets and to worsen glucose tolerance in vivo through NLRP3‐inflammasome‐linkeddisruption of calcium handling, Serca2 loss, ER stress, *β*‐cell dedifferentiation, and apoptosis [31, 32]. Together, these data support a model in which the diabetic microbiome contributes not only to peripheral insulin resistance but also to progressive *β*‐cell dysfunction.

These mechanistic insights have important translational consequences. First, they argue that microbiome‐informed phenotyping in diabetes should move beyond broad compositional readouts and instead incorporate functional measures such as SCFA‐generating capacity, bile‐acid signatures, and circulating harmful metabolites such as ImP and TMAO. Second, they support more selective intervention strategies. Patients with low fermentative capacity may preferentially benefit from dietary fiber, resistant starch, or synbiotic approaches, whereas patients with high‐risk metabolite signatures may ultimately require more specific microbiome‐targeted therapeutics, including inhibitors of harmful microbial enzymes [40]. Third, translational microbiome manipulation is now moving past short‐term proof‐of‐concept. In obese subjects with T2DM, microbiota engraftment after FMT has been demonstrated in a 24‐week RCT [41], and longer term follow‐up work in adolescents with obesity has shown sustained donor‐derived bacterial and bacteriophage persistence together with improvements in waist circumference, total body fat, metabolic syndrome severity, HDL cholesterol, and hs‐CRP despite no major BMI effect [42]. Meta‐analytic evidence also suggests that FMT may improve selected measures of body weight, insulin resistance, and glycemic control in obesity‐associated metabolic disease, although differences in donor selection, administration protocols, concomitant lifestyle interventions, and study populations complicate direct comparison across trials [43]. Collectively, these findings suggest that future diabetes management may need to classify patients not only by glycemic phenotype but also by microbial function and metabolite output.

### 4.2. IBD and IBS

Microbiota‐derived metabolites are equally central to gastrointestinal disease, but their effects are expressed locally through disease‐specific epithelial, immune, sensory, and secretory circuits. Recent work emphasizes that the microbiome in IBD should be understood not simply as a descriptive change in bacterial abundance, but as a cross‐kingdom ecological and metabolic system capable of shaping inflammation, intestinal biology, pain pathways, and therapeutic responsiveness through bacteria‐, fungi‐, and metabolite‐dependent mechanisms [44]. From this perspective, the key question is not only whether dysbiosis is present, but also which microbial functions and metabolites are depleted or enriched, and how those changes are translated into chronic mucosal or functional gastrointestinal disease.

In IBD, one of the most consistent metabolite‐level defects is the loss of SCFA support to the colonic epithelium. Butyrate is the preferred oxidative substrate for colonocytes, and defective butyrate utilization has been documented in inflamed mucosa, indicating that SCFA deficiency is not merely an associative marker of dysbiosis but a direct contributor to impaired epithelial energetics [44]. SCFAs also reinforce barrier integrity, regulate gene expression through histone deacetylase inhibition, and support mucosal homeostasis through receptor‐mediated and epigenetic mechanisms [33, 37]. Thus, depletion of butyrate‐producing communities in IBD is important because it removes both an epithelial fuel source and an anti‐inflammatory metabolic signal required for tissue restitution.

Bile‐acid dysmetabolism provides a second major mechanistic axis in IBD. Contemporary work now places BAs at the center of host–microbiome metabolic interactions with relevance to intestinal inflammation and therapeutic targeting [45, 46]. Primary studies have shown that specific microbiota‐derived bile‐acid metabolites regulate *T* helper 17 and regulatory T‐cell differentiation and that disruption of these pathways can alter mucosal immune tone [47–49]. Consistent with this framework, dysbiosis‐induced secondary bile‐acid deficiency has been shown to promote intestinal inflammation [50]. Recent systematic evidence further supports bile‐acid dysmetabolism as a feature of IBD. A systematic review of studies in Crohn’s disease identified recurring alterations in bile‐acid profiles, including reductions in key secondary BAs, reinforcing the potential relevance of microbiota‐dependent bile‐acid metabolism to intestinal inflammation [51]. These observations are highly relevant to the present review because they show how altered microbial biotransformation of BAs can influence epithelial signaling, adaptive immunity, and inflammatory persistence in IBD. This also strengthens the translational argument for incorporating bile‐acid profiling and metabolite‐centered microbiome analysis into future IBD stratification. Therapeutic manipulation of the intestinal microbiota is also being investigated directly in IBD. A 2025 systematic review and meta‐analysis of RCTs found that FMT can promote clinical and endoscopic remission in ulcerative colitis, supporting the therapeutic relevance of microbiome modification while underscoring the need to optimize donor selection, treatment protocols, and patient stratification [52]. Importantly, therapeutic response may depend on specific ecological characteristics rather than microbiota transfer alone. A recent systematic review and meta‐analysis linked favorable FMT outcomes in ulcerative colitis with greater microbial diversity and the presence of several butyrate‐producing taxa, suggesting that microbial features may eventually help guide donor selection and personalized microbiota‐based therapy [53].

IBS should also be expanded beyond a generic dysbiosis paragraph. Functional and metagenomic studies indicate that IBS shares some compositional and functional microbiome alterations with IBD while remaining pathophysiologically distinct [54]. A major translational concept is the existence of a bile‐acid‐driven IBS phenotype, especially in diarrhea‐predominant disease, in which excessive delivery of primary BAs to the colon can increase water secretion, accelerate transit, and amplify symptoms. Recent work on BA–GM crosstalk in IBS underscores that altered microbial transformation of BAs is not only descriptive but can define clinically meaningful endotypes with implications for diagnosis and treatment [55]. Beyond BAs, IBS can also be framed through abnormal metabolite‐dependent gut–brain and enteroendocrine signaling. SCFAs and indole derivatives influence GLP‐1 and PYY release, epithelial activity, and immune signaling through pathways involving FFAR2/FFAR3, HDAC inhibition, AhR, and PXR, thereby providing plausible mechanisms through which altered microbial metabolism may influence visceral hypersensitivity, motility, symptom fluctuation, and food‐related responses [33]. Supporting the clinical relevance of this pathway, a 2024 systematic review and meta‐analysis identified alterations in fecal SCFA profiles in IBS and evaluated their modification by therapeutic interventions, further linking microbial metabolic output with IBS pathophysiology and treatment response [56]. This mechanistic framing is especially valuable because it links microbiota‐derived chemistry to hallmark IBS features such as altered transit, meal‐triggered symptoms, and gut–brain axis dysfunction, rather than reducing IBS to the inconsistent presence or absence of specific taxa.

Taken together, IBD and IBS illustrate that gastrointestinal disorders are not merely “associated” with the microbiome; they are shaped by disease‐specific disruptions in microbial metabolism. Clinical efforts to manipulate this ecosystem have nevertheless produced heterogeneous results. A recent systematic review and meta‐analysis of probiotic trials in IBS found evidence of benefit for some preparations and outcomes while also highlighting substantial variation among probiotic strains, combinations, trial designs, and endpoints [57]. In IBD, the dominant consequences are epithelial energy deficiency, secondary bile‐acid loss, and maladaptive immune activation; in IBS, the dominant consequences are altered secretion, motility, sensory signaling, and neuroendocrine responses. Translationally, this means that future gut‐directed therapy is likely to become more mechanism‐based and less empiric, incorporating diet‐based metabolic modulation, biomarker‐guided bile‐acid phenotyping, postbiotic or metabolite replacement strategies, and selective targeting of harmful microbial pathways rather than indiscriminate manipulation of overall community composition.

## 5. Modulating the Microbiota: Therapeutic Interventions and Evidence

The growing mechanistic understanding of the microbiota–metabolism axis has spurred the development of therapeutic strategies aimed at correcting functional dysbiosis. These interventions range from broad dietary changes to highly specific microbial transplantation, with a growing body of clinical and preclinical evidence supporting their potential Table 1. At the clinical level, a recent systematic review and meta‐analysis of 41 RCTs found that microbiota‐targeted interventions, including probiotics, prebiotics, dietary approaches, and FMT, were associated with improvements in multiple glucose and lipid parameters, although responses differed by intervention type and duration [58].

**TABLE 1:** Summary of clinical and preclinical interventions targeting the gut microbiota in metabolic disease.

Intervention	Study design/model	Key microbial and metabolite changes	Primary metabolic outcomes	Citation
Legume‐enriched diet	16‐week RCT in prediabetic adults	↑ Fiber‐degrading bacteria; altered BAs and amino acid derivatives	↓ HbA1c, ↓ LDL/total cholesterol	[38]
Fecal microbiota transplantation (FMT) + lifestyle intervention	24‐week double‐blind RCT in obese T2DM	Successful engraftment of lean donor microbiota; ↑ Butyrate producers, ↑ *Bifidobacterium*, *Lactobacillus*	↓ LDL cholesterol, ↓ Liver stiffness	[41]
Targeted acetate delivery	8‐week study in diabetic db/db mice	↑ Colonic and circulating acetate	Improved glucose tolerance; ↑ Circulating GLP‐1 and PYY	[22]
Metformin vs. ImP	Mouse model and human correlational study	Focus on metabolite interaction	ImP administration blunts metformin’s glucose‐lowering effect in mice. High ImP correlates with poor metformin response in humans	[30]

*Note:* PYY, peptide YY; ImP, imidazole propionate; HbA1c, glycated hemoglobin.

Abbreviations: BA, bile acids; FMT, fecal microbiota transplantation; GLP‐1, glucagon‐like peptide‐1; LDL, low‐density lipoprotein; RCT, randomized controlled trial; T2DM, Type 2 diabetes mellitus.

### 5.1. Dietary and Prebiotic Strategies

Diet remains the most potent and accessible tool for shaping the gut microbiome and its metabolic output. Strategies focused on increasing the intake of fermentable fibers are designed to boost the populations of beneficial, SCFA‐producing bacteria. High‐quality evidence for this approach comes from a recent RCT by Wu et al. [38]. In this 16‐week study, adults with prediabetes who consumed a calorie‐restricted diet enriched with legumes demonstrated significantly greater improvements in HbA1c and LDL cholesterol compared to a group on a calorie‐matched control diet. Advanced multi‐omics analysis directly linked these superior metabolic outcomes to favorable shifts in the gut microbiome’s composition and function, including changes in BA and amino acid derivative profiles. This study provides strong human evidence that targeted dietary interventions can ameliorate metabolic disease by positively modulating the microbiome. This translational potential is further supported by a recent systematic review and network meta‐analysis showing that probiotics, prebiotics, and synbiotics can improve several indices of glycemic control in patients with T2DM, although the magnitude of benefit varies according to intervention type and formulation [59].

### 5.2. Drug–Microbiome Synergy and Interference

As previously discussed, microbiomes can significantly influence the efficacy of pharmacotherapies. The interaction between metformin and ImP, described by Koh et al. (2020), serves as a clear example of microbial interference, where a specific metabolite directly antagonizes a drug’s mechanism of action [30]. Conversely, some of metformin’s own therapeutic benefits are now thought to be mediated through its effects on the GM, such as promoting the growth of SCFA‐producing bacteria and *Akkermansia muciniphila* [60]. This bidirectional relationship underscores the critical need to incorporate pharmacomicrobiomic principles into drug development and clinical practice.

### 5.3. FMT

FMT represents the most direct approach to remodeling a dysbiotic microbial community by introducing a complete, healthy ecosystem from a screened donor. While highly effective for *Clostridioides difficile* infection, its application in metabolic disease has yielded more variable results. A landmark double‐blind, RCT by Ng et al. (2022) provided crucial insights into optimizing FMT for T2DM [41]. The study showed that repeated FMT from healthy lean donors could achieve successful and durable engraftment of beneficial microbes in obese individuals with T2DM. However, the most significant clinical benefits—namely, reductions in LDL cholesterol and liver stiffness (a marker of nonalcoholic fatty liver disease)—were observed in the group that received FMT in combination with a structured lifestyle intervention. This finding supports a “seed and soil” model for microbiota therapeutics [41]. The FMT provides the beneficial microbes (the “seed”), but the host’s dietary and physiological environment (the “soil”) must be conducive to their growth and function. The lifestyle intervention, likely by providing the necessary fiber substrates, created a favorable environment for the transplanted SCFA‐producing bacteria to thrive and exert their metabolic benefits. At the evidence‐synthesis level, a recent systematic review and meta‐analysis of randomized trials similarly found that FMT can modify gut microbial composition and may improve selected metabolic outcomes in T2DM; however, the limited number of available trials and substantial methodological heterogeneity indicate that larger standardized studies are still needed [61]. This suggests that future microbiota‐based therapies will be most effective when they are integrated into a holistic approach that supports the engraftment and long‐term stability of the desired microbial functions.

## 6. Clinical and Translational Outlook: Toward Precision Medicine

The continued elucidation of these mechanistic pathways is paving the way for a new era of precision medicine in metabolic and gastrointestinal health. The focus is shifting from one‐size‐fits‐all approaches to personalized strategies guided by an individual’s unique microbial and metabolic profile. Recent systematic reviews and translational studies reinforce this shift while also demonstrating that clinical responses to microbiota‐directed interventions are highly heterogeneous, emphasizing the need for standardized protocols, functional biomarkers, and biologically informed patient stratification [36, 53, 58].

A key development will be the use of microbial metabolites as clinical biomarkers. Rather than relying on complex and expensive metagenomic sequencing, measuring the circulating levels of key functional readouts like ImP, TMAO, specific BA ratios, or even SCFAs could provide a more direct, cost‐effective, and actionable assessment of a patient’s metabolic risk profile [35]. Such biomarkers could be used to stratify patients for early and aggressive preventive interventions or to guide therapeutic choices. For example, a high ImP level could identify an individual at high risk for T2DM who might benefit most from a targeted dietary intervention and who may respond poorly to metformin. Building on this biomarker‐driven approach, the future of treatment lies in precision nutrition and microbiota‐directed therapeutics. This could involve the development of next‐generation “synbiotics,” which combine specific probiotic strains with tailored prebiotic fibers designed to maximize their beneficial metabolic output [62]. Another promising avenue is the development of small‐molecule drugs that specifically inhibit the microbial enzymes responsible for producing pathogenic metabolites like ImP or TMA [40]. By targeting the function rather than the organism, such therapies could selectively silence the detrimental activities of the microbiome while preserving its overall structure and beneficial functions.

## 7. Limitations and Future Directions

Despite rapid progress, several challenges and limitations remain in the field. A primary hurdle is the difficulty of establishing definitive causality in human studies. While animal models provide powerful mechanistic proof, confirming these causal links in complex human systems is challenging due to confounding variables like diet, genetics, and lifestyle. Furthermore, the long‐term durability of interventions like diet and FMT needs to be rigorously assessed in studies with extended follow‐up periods to determine whether the induced microbial and metabolic changes are sustained [42, 63, 64].

There is also a pressing need for the standardization of methodologies across research studies. Variations in sample collection, storage, processing, and data analysis pipelines make it difficult to compare results and perform meta‐analyses. Overcoming the high degree of inter‐individual variability in both baseline microbiome composition and response to interventions will require larger, more diverse, and deeply phenotyped clinical trials [65]. Future research should focus on identifying the host and microbial factors that predict response, allowing for the stratification of patients into likely responders and nonresponders to a given microbiota‐targeted therapy.

## 8. Conclusion

The GM has unequivocally emerged as a central regulator of host metabolism and a key player in the pathogenesis of T2DM and related gastrointestinal disorders. Its influence is exerted through a sophisticated chemical dialog mediated by a diverse suite of bioactive metabolites. A functional balance, characterized by robust SCFA production from dietary fiber and well‐regulated BA signaling, promotes metabolic health by enhancing incretin secretion and insulin sensitivity. Conversely, a state of functional dysbiosis, marked by a deficit of these beneficial signals and an overproduction of pathogenic metabolites like ImP and TMAO, actively drives insulin resistance and *β*‐cell failure. This integrated perspective, linking microbial function to host physiology, is transforming our approach to metabolic disease. Targeting the microbiota–metabolism axis through rational, evidence‐based interventions—from precision nutrition and next‐generation biotherapeutics to pharmacomicrobiomic‐guided drug selection—holds immense promise for the future prevention and management of these chronic conditions, heralding a new era of metabolically informed medicine.

**Nomenclature**
AMPK: AMP‐activated protein kinase
BA: Bile acid
BAs: Bile acids
β‐cell: Pancreatic beta cell
DB: Diabetes
ER: Endoplasmic reticulum
FFAR2 (GPR43): Free fatty acid receptor 2
FFAR3 (GPR41): Free fatty acid receptor 3
FMT: Fecal microbiota transplantation
FXR: Farnesoid *X* receptor
GLP‐1: Glucagon‐like peptide‐1
GLP‐1RA: GLP‐1 receptor agonist
GM: Gut microbiota
GSIS: Glucose‐stimulated insulin secretion
GPR: G‐protein‐coupled receptor
IBD: Inflammatory bowel disease
IBS: Irritable bowel syndrome
ImP: Imidazole propionate
IRS1: Insulin receptor substrate‐1
L‐cell: Enteroendocrine L‐cell
mTORC1: Mechanistic target of rapamycin complex 1
NAFLD: Non‐alcoholic fatty liver disease
NLRP3: NOD‐, LRR‐ and pyrin domain‐containing protein 3
PYY: Peptide YY
RCT: Randomized controlled trial
SCFA: Short‐chain fatty acid
SCFAs: Short‐chain fatty acids
T2DM: Type 2 diabetes mellitus
TGR5: Takeda G‐protein‐coupled receptor 5
TMAO: Trimethylamine N‐oxide
TMA: Trimethylamine
WSCA: Water‐soluble cellulose acetate

## Funding

No funding was received for this manuscript.

## Conflicts of Interest

The authors declare no conflicts of interest.

## Data Availability

Data sharing is not applicable to this article as no datasets were generated or analyzed during the current study.
